# Patient satisfaction with doctor-patient interaction and its association with modifiable cardiovascular risk factors among moderately-high risk patients in primary healthcare

**DOI:** 10.7717/peerj.2983

**Published:** 2017-02-21

**Authors:** Mohd Noor Norhayati, Abd Aziz Masseni, Ishak Azlina

**Affiliations:** 1 Department of Family Medicine, Universiti Sains Malaysia, Kubang Kerian, Kelantan, Malaysia; 2 Klinik Kesihatan Menggatal, Kota Kinabalu, Sabah, Malaysia

**Keywords:** Patient satisfaction, Doctor-patient interaction, Medical interview satisfaction scale-21, Primary healthcare, Cardiovascular risk

## Abstract

**Background:**

The outcomes of the physician-patient discussion intervene in the satisfaction of cardiovascular disease risk patients. Adherence to treatment, provision of continuous care, clinical management of the illness and patients’ adjustment are influenced by satisfaction with physician-patient interaction. This study aims to determine the patient satisfaction with doctor-patient interaction and over six months after following prevention counselling, its associations with modifiable cardiovascular risk factors amongst moderately-high risk patients in a primary healthcare clinic in Kelantan, Malaysia.

**Methods:**

A prospective survey was conducted amongst patients with moderately-high cardiovascular risk. A total of 104 moderately-high risk patients were recruited and underwent structured prevention counselling based on the World Health Organization guideline, and their satisfaction with the doctor-patient interaction was assessed using ‘Skala Kepuasan Interaksi Perubatan-11,’ the Malay version of the Medical Interview Satisfaction Scale-21. Systolic blood pressure, total cholesterol and high-density lipoprotein cholesterol were measured at baseline and at a follow-up visit at six months. Descriptive analysis, paired *t* test and linear regression analyses were performed.

**Results:**

A total of 102 patients responded, giving a response rate of 98.1%. At baseline, 76.5% of the respondents were satisfied with the relation with their doctor, with the favourable domain of distress relief (85.3%) and rapport/confidence (91.2%). The unfavourable domain was interaction outcome, with satisfaction in only 67.6% of the respondents. Between the two visits, changes had occurred in total cholesterol (*P* = 0.022) and in systolic blood pressure (*P* < 0.001). Six months after the initial visits, no relationship existed between patient satisfaction scores and changes in modifiable cardiovascular risks.

**Discussion:**

The ‘Skala Kepuasan Interaksi Perubatan-11’ which represents a component of the interpersonal doctor-patient relationship can be used to assess improvements of the medical skills and in medical training to enhance the quality of therapeutic communication.

## Introduction

Globally, in 2005, cardiovascular disease (CVD) accounted for 30% of an estimated 58 million deaths (WHO, 2005) and deaths due to non-communicable diseases, half of which is due to CVD, are expected to increase by 17% between 2006 and 2015 (WHO, 2005). Public health approaches alone, targeting on wide population, will not have an immediate tangible impact on cardiovascular morbidity and mortality (Manuel et al., 2006a). The cardiovascular disease burden could possibly be reduced by a combined strategies on population-wide and high-risk individuals, emphasizing on achievable effectiveness, cost-effectiveness and available resources (Lopez et al., 2006; Manuel et al., 2006b; WHO, 2002; WHO, 2005).

Satisfaction is the perception of an individual’s experience compared with his or her expectations. For patients, it is related to the extent to which general health care needs and condition-specific needs are met (Pascoe, 1983). For every patient, medical consultation forms part of the strategies to deal and cope with illness. Evaluating to what extent patients are satisfied with health services is clinically relevant, as satisfied patients are more likely to comply with treatment (Guldvog, 1999).

Patients’ satisfaction of their relation with their doctors is a key element in the efficiency and usage of health services and varies depending on patient characteristics (Arber et al., 2006). Each patient has expectations when meeting a doctor and the difference between these expectations and what he obtains represent the perception of the satisfaction (Abioye Kuteyi et al., 2010). Adherence to treatment, provision of continuous care, clinical management of the illness and patients’ adjustment are very closely related to satisfaction with physician-patient interaction (Loblaw, Bezjak & Bunston, 1999). Satisfied patients described their primary care doctors as showing genuine interest in their health problems, able to provide clear descriptions of the diseases and future health consequences, gave them ample opportunities to talk about health and how the disease affected their everyday life (Platonova & Shewchuk, 2015).

The Consultation Satisfaction Questionnaire (CSQ) and Medical Interview Satisfaction Scale (MISS) are two tools used to measure satisfaction that have been validated and found to be reliable (Anden, Andersson & Rudebeck, 2006; Kinnersley et al., 1996). A study comparing MISS-29 and the CSQ showed neither questionnaire was superior to the other in psychometric terms. Both have high level of internal consistency. However, MISS measures humaneness more consistently than CSQ (Kinnersley et al., 1996). The MISS-21 questionnaire has been used in several studies to measure patient satisfaction (Abioye Kuteyi et al., 2010; Singh, Haqq & Mustapha, 1999; Van Uden et al., 2005). MISS-21 is a self-administered questionnaire exploring the four domains of doctor-patient interaction, namely information provision by the doctor (distress relief; six items), patient’s confidence in the doctor (rapport; eight items), doctor’s communication skills (communication comfort; three items) and adherence intent (compliance intent; four items) (Meakin & Weinman, 2002).

The focus regarding CVD prevention and information sharing with patients have shifted from individual risk factors (example, hypertension or hyperlipidaemia) to global (multiple) risk factors (Krones et al., 2008). Assessment of cardiovascular risk is important for attending physicians and other healthcare personnel to identify patients with an elevated 10-year risk of experiencing coronary heart disease events so that potential problems can be addressed earlier via adapted treatments or other interventions (Grundy et al., 2004). The Framingham Risk Assessment tools have been used extensively with men and women and with a number of ethnic groups and are considered the ‘gold standard’ for risk assessment (Coke, 2010). Risk factors used in Framingham scoring include age, total cholesterol (TC), high-density lipoprotein cholesterol (HDL), blood pressure and cigarette smoking. The Framingham scoring tool divides persons with multiple risk factors according to 10-year risk for coronary heart disease (CHD) into the risk categories of >20%, 10–20% middle and <10% (National Institutes of Health, 2001).

For this study, we choose the traditional Framingham risk score because this has been used worldwide since 1998 and is being validated across populations i.e., men, women, blacks, European, Mediterranean and Asian (Berger et al., 2010; Chia & Srinivas, 2010). Recently, this score (using low equations) was used to compare data from Framingham study with Asian cohort on the accuracy of cardiovascular risk prediction; resulted in similar accuracy in Asian population after recalibrated (Barzi et al., 2007). Currently, no standard modules or decision aids are used for counselling on cardiovascular risk in a primary healthcare setting in Kelantan, Malaysia. Kelantan is a state in the northeast of Peninsular Malaysia with a population approximately 1.7 million. Approximately 32% of the population lives in Kota Bharu, the capital city of Kelantan. Systematic review of CHD showed that the quality of educational interventions in risk can improve compliance (Ham, 2010).

This study aims are: first, to analyse the patient satisfaction on doctor-patient interaction following CVD risk prevention counselling based on the World Health Organization guideline (WHO, 2007); second, to determine the association between patient satisfaction scores and changes in modifiable cardiovascular risk factors among moderately high-risk patients.

## Materials & Methods

### Study design and sample

A six-month prospective survey was conducted among moderately-high CV risk patients in a primary outpatient care of a tertiary hospital in Kota Bharu from 2012 to 2013. We included: moderately-high risk patients with multiple (2 or more) risk factors and a 10-year risk for CHD of 10–20% (National Institutes of Health, 2001). The risk factors are cigarette smoking, hypertension (BP ≥ 140/90 mmHg or on antihypertensive medication), low-density lipoprotein cholesterol (LDL) (<1.1 mmol/L), family history of premature CHD (CHD in a male first-degree relative at <55 years, or in a female first-degree relative at <65 years) and age ≥45 years for men and ≥55 years for women. We excluded illiterate patients and those with established CHD or a history of stroke.

Sample size calculation was based on the first objective i.e., patient satisfaction on doctor-patient interaction. Based on single-proportion formula, the proportion of patients intending to adhere to treatment, a component of patient satisfaction, was 82.7% (Abioye Kuteyi et al., 2010). Using the precision of 0.08 and 95% confidence, the minimum required sample size was 86; after considering the non-response rate of 20%, the calculated sample size was determined to be 104. All of these patients fulfilling the eligibility criteria were invited to participate in the study.

### Ethical consideration

The study protocol was approved by the Human Research Ethics Committee, Universiti Sains Malaysia (USMKK/PPP/JePeM[245.3.(9)]) on 6 February 2012. Patients were provided information related to the study along with an Information and Consent Form. Confidentiality was ensured and written informed consent was obtained.

### Data collection

Patients attending the primary healthcare clinic were screened for the cardiovascular risk factors based on their medical records. Risk factors were calculated using the Framingham risk factors point score and 10-year specific risk by gender. These Framingham risk scores, delineated in Adult Treatment Panel III (ATP III), have been used since 2001 and consist of five risk scores (National Institutes of Health, 2001).

Prior to the day of the visit, patients were asked to fast overnight. On the day of the visit, 3 ml of blood were taken from each patient and sent for laboratory testing to determine fasting cholesterol levels. Also obtained during the visit were body mass index (BMI) (kg/m ^2^), waist circumference (WC) (cm), and blood pressure (mm Hg; measured with a calibrated standard digital blood pressure machine (Omron Global, Kyoto, Japan).

A structured intervention counselling with a standardize content for CVD prevention was carried out by the researcher; this ensured that there was limited bias in measuring patient satisfaction with the doctor-patient interaction. After counselling, patients were then required to answer the Malay version of the MISS-21 questionnaire outside the consultation room immediately after counselling. Upon completion, patients submitted the questionnaire to the research assistant. Six months after their initial visits, patients returned for follow-up appointments with the same healthcare practitioner, during which 3 ml of blood were drawn to obtain a fasting lipid profile (TC, HDL, LDL); blood pressure was also measured.

### Research tool

*Skala Kepuasan Interaksi Perubatan (SKIP-11)* is a modification of the Malay version of MISS-21 suggested that the three-factor model with 11 items, acceptable for use in assessing patient satisfaction with patient-physician interaction in a primary healthcare setting because it is valid, reliable and simple. The three domains are as follows: (i) information provision by the doctor (distress relief; four items), (ii) patient’s confidence in doctor (rapport; four items) and (iii) doctor-patient interaction outcome (interaction outcome; three items). Cronbach’s alpha was 0.513 for distress relief, 0.708 for rapport and 0.747 for interaction outcome. The overall Cronbach’s alpha was 0.669, indicating a satisfactory level of internal consistency. The patients’ subscale scores were added together and the mean subscale scores were determined (Abd Aziz et al., 2013). Categorisation into satisfied and dissatisfied categories was based on the overall items and items for each subscale. Score >44 for overall items and score >16 for distress relief, >16 for rapport and >12 for interaction outcome subscales are considered as satisfied.

*CVD risk prevention counselling* applied a risk-stratification approach that covered adherence to medication and advice on smoking cessation, healthy dietary choices, physical activity and weight reduction based on recommended steps to help prevent cardiovascular disease by the WHO (2007).

*Framingham cardiovascular risk assessment* provides separate score sheets for men and women. The scores for age, total cholesterol, high density lipoprotein cholesterol, blood pressure and cigarette smoking are summed up and the 10-year coronary risk is determined. Patients are then divided into those with 10-year risk for CHD of >20%, 10–20%, and <10%. Moderately-high risk is used to describe individuals with multiple (two or more) risk factors and a 10-year risk for CHD of 10–20% (National Institutes of Health, 2001).

### Data analyses

Data checking and cleaning were performed prior to data entry and analysis using SPSS version 19. Descriptive analysis was used to determine the overall patient satisfaction score and subscale of distress relief, confidence rapport and doctor-patient interaction outcome. Paired *t* test were used to determine the change in modifiable cardiovascular risk factors i.e., TC (mmol/L), HDL (mmol/L), and systolic blood pressure (SBP) (mmHg) that were based on risk factors used in the original Framingham scoring system (National Institutes of Health, 2001) between the appointments at baseline and six-month follow-up. Simple and Multiple linear regression confirmatory analyses were used to determine the association between patient satisfaction scores and modifiable risk factors (TC, HDL, SBP). Score changes in TC, HDL, and SBP served as the outcome variables. The fixed factor was patient satisfaction score; the adjusted variables were sociodemographic (age, gender, level of education) and medical (smoking status; presence of diabetes; presence of hypertension; baseline BMI, WC, SBP, TC, and HDL). General linear regression confirmatory analysis was used to examine the relationship between one numerical dependent variable and more than one independent variable with the goal to develop a best fitting, parsimonious, biologically sound and easy model to estimate the beta.

## Results

A total of 104 moderately-high risk patients attending primary healthcare clinic were recruited and 102 respondents were included in the analyses (a response rate of 98.1%). Of the two who did not respond, one could not be contacted and the other had died of cancer at the time of the six-month follow-up appointment. Table 1 provides the sociodemographic and medical characteristics of 102 respondents at moderately-high cardiovascular risk.

**Table 1:** Sociodemographic and medical characteristics of 102 respondents.

Variables	Mean	(SD^a^)	*n*	(%)
***Socio-demographic characteristics***				
Age (years)	60.1	(7.59)		
Sex				
Male			74	(72.5)
Female			28	(27.5)
Race				
Malay			85	(83.3)
Non-Malay			17	(16.7)
Education level				
Primary school			22	(21.6)
Secondary school			50	(49.0)
College and university			30	(29.4)
Marital status				
Yes			100	(98.0)
No			2	(2.0)
Smoking status				
No			83	(81.4)
Yes			19	(18.6)
Hypertension				
No			5	(4.8)
Yes			97	(95.2)
Diabetes mellitus				
No			65	(63.7)
Yes			37	(36.3)
Family history of cardiovascular disease				
No			101	(99.0)
Yes			1	(1.0)
***Medical characteristics***				
Body mass index (kg/m ^2^)	27.0	(4.66)		
Waist circumference (cm)^b^	93.0	(14.76)		
Systolic blood pressure (mmHg)	146.4	(14.28)		
Diastolic blood pressure (mmHg)	88.1	(10.44)		
Total cholesterol (mmol/L)	5.6	(1.34)		
High-density lipoprotein cholesterol (mmol/L)^c^	1.2	(0.36)		
Low-density lipoprotein cholesterol (mmol/L)	3.5	(1.20)		

**Notes.**

a Standard deviation.

b Three values are missing.

c Median (interquartile range); data are skewed to the right.

Based on SKIP-11, 78 respondents (76.5%) were satisfied with the doctor-patient interaction. The total patient satisfaction scores were normally distributed ranging from 38 to 55 with a mean (SD) of 47.6 (4.40). The mean (SD) and percentage of respondents satisfied with the doctor-patient interaction according to the SKIP-11 subscales are shown in Table 2.

**Table 2:** Subscales and total scales of patient satisfaction as measured with SKIP-11.

Subscale	Score	Mean	(SD^a^)	*n*	(%)
Distress relief	>16	17.5	1.72	87	85.3
Rapport/confidence	>16	17.9	1.80	93	91.2
Interaction outcome	>12	12.2	2.04	69	67.6
SKIP-11	>44	47.6	4.40	78	76.5

**Notes.**

SKIP 11 Skala Kepuasan Interaksi Perubatan

a Standard deviation.

Table 3 shows the change in modifiable cardiovascular risk factors (TC, HDL, SBP) between baseline and six-month follow-up. Both SBP (*P* < 0.001) and TC (*P* = 0.022) significantly decreased post-intervention at six-month follow-up.

**Table 3:** Change in modifiable cardiovascular risk factors from baseline to 6-month follow-up.

CVD risks	Baseline mean (SD^a^)	6-month EMM^b^ (SD^a^)	Mean diff (95% CI)^c^	*t* stat^d^	*P* value
SBP (mm Hg)	146.35 (14.28)	133.84 (10.68)	12.51 (9.69, 15.33)	8.80	<0.001
TC (mmol/L)	5.58 (1.34)	5.28 (1.19)	0.30 (0.04, 0.55)	2.33	0.022
HDL (mmol/L)	1.25 (0.38)	1.21 (0.28)	0.04 (0.03, 0.12)	1.19	0.236

**Notes.**

SBP systolic blood pressure TC total cholesterol HDL high-density lipoprotein cholesterol

a Standard deviation.

b Estimated marginal mean.

c Mean difference (95% confidence interval).

d *t* statistic.

Simple linear regression analyses showed no association between patient satisfaction score and changes in modifiable cardiovascular risk factors such as TC (*P* = 0.886), HDL (*P* = 0.077), and SBP (*P* = 0.457). General linear regression analyses also showed no association between patient satisfaction score and changes in modifiable cardiovascular risk factors such as TC (*P* = 0.457), HDL (*P* = 0.070), and SBP (*N* = 0.917) when controlling for potential confounders in the model (Table 4). Residual plots indicate that overall model fitness, the equal variance assumption, the normality assumption and variable functional forms were satisfied. No outliers occurred when plotting studentised residuals against the predicted value. There is no association between patient satisfaction score and modifiable cardiovascular risk factors (TC, HDL, SBP) after controlling for potential confounders as shown in Table 4.

**Table 4:** Association between patient satisfaction scores and changes in modifiable cardiovascular risk factors.

CVD	SLR^a^			GLR^b^		
Risks	*b*^c^ (95% CI^d^)	*t* stat^e^	*P* value	Adj b^f^ (95% CI^d^)	*t* stat^e^	*P* value
TC^g^	0.004 (0.058, −0.050)	0.14	0.886	0.019 (0.069, −0.031)	0.75	0.457
HDL^h^	−0.011 (0.001, −0.024)	−1.79	0.077	−0.011 (0.001, −0.024)	−1.83	0.070
SBP^i^	0.181 (0.662, −0.299)	0.75	0.457	0.025(0.493, −0.444)	0.10	0.917

**Notes.**

TC total cholesterol  HDL high-density lipoprotein cholesterol SBP systolic blood pressure

a Simple linear regression.

b General linear regression. There was no interaction between the fixed factor and the controlled variables and no multicollinearity problem; model assumptions met.

c Crude regression coefficient.

d Confidence Interval.

e *t* statistic.

f Adjusted regression coefficient.

g *R*^2^ of GLR was 33.1% after adjustment for age, sex, education level, presence of DM, presence of HPT, baseline BMI, WC, SBP, TC and HDL.

h *R*^2^ of GLR was 33.2% after adjustment for age, sex, education level, presence of DM, presence of HPT, baseline BMI, WC, SBP, TC and HDL.

i *R*^2^ of GLR was 31.0% after adjustment for age, sex, education level, presence of DM, presence of HPT, baseline BMI, WC, TC, HDL and SBP.

## Discussion

### Patients’ satisfaction of the relation with their doctor

Using the ‘Skala Kepuasan Interaksi Perubatan-11’ Malay version of MISS-21, our findings revealed that about three-quarters of the patients were satisfied with their experience in primary healthcare clinic with their doctor-patient interaction. It was consistent with other studies that evaluated patient satisfaction in primary healthcare clinic, where 63.3% were satisfied in a study in Nigeria (Abioye Kuteyi et al., 2010), 74% in Trinidad and Tobago (Singh, Haqq & Mustapha, 1999) and 81% in the Netherlands (Van Uden et al., 2005). With regards to method of assessment for patients’ satisfaction, only Abioye Kuteyi et al. (2010) used MISS. Singh, Haqq & Mustapha (1999) used self-developed questionnaire to assess patient satisfaction with three groups of healthcare workers, namely doctors, nurses and pharmacists. About 74% of the patients were satisfied with doctors (Singh, Haqq & Mustapha, 1999), which was comparable with the present study. In another study, the CSQ was mailed to patients three weeks after consultation followed by telephone interview by the nurses (Van Uden et al., 2005). The satisfaction concerning visits to general practice (81%) was available in only one study (Van Uden et al., 2005) and the findings were comparable.

About 91% of our study patients were satisfied with the physician confidence rapport domain and 85% were satisfied with the distress relief domain; this was in line with a previous review (Williams, Weinman & Dale, 1998). Our major findings showed that the domain of distress relief was favourable among patients, and a good rapport was observed between the doctor and patient. Patient satisfaction was associated with doctor-patient interaction, specifically the patient’s confidence, perception of the doctor’s communication skills and perception of information provision. Meanwhile, adherence intent was related with patient’s confidence and perception of information provision by the doctor (Abioye Kuteyi et al., 2010).

Another interesting finding, about 24% of our study patients were not satisfied with the doctor-patient interaction using similar cut-off score for satisfaction. In other studies, this rate were 26% (Singh, Haqq & Mustapha, 1999) and 36% (Abioye Kuteyi et al., 2010) of the patients were not satisfied with the doctor-patient interaction. One study reported satisfaction as mean scores with higher scores indicating more satisfied (Van Uden et al., 2005).

Among our patients, 33% of them were not satisfied with the interaction outcome domain and, as a result, felt uneasy or unsure about following the advice given by the physician. The causes of unfavourable findings in this domain could be due to patients’ unwillingness to change their behaviour and adhere to the advice. A study by Berra (2003) on the Health Education and Risk Reduction Training Program (HEAR2T), which combined intensive behavioural risk factor control and medical therapies, showed that patient satisfaction towards the counselling given was very good (97%). However, only 69% of the patients that reported they were satisfied made behavioural changes, and 73% making behavioural changes expressed that they were confident in their ability to maintain these changes or adherence (Berra, 2003).

### Association between patients’ satisfaction and cardiovascular risk factors

After six-month follow-up, our primary prevention counselling showed a relevant beneficial decrease in various modifiable cardiovascular risk factors (namely, TC and SBP). But reductions in SBP and TC were not related to the level of patient satisfaction and did not contribute to patient outcomes.

Satisfaction with the patient-physician interaction on the part of the patient does not signify that the patient will adhere to a medication; the converse is also true. Satisfied patients (63.3%) were more likely to adhere to advice from physicians (94.2%) but 56.6% of unsatisfied patients (36.7%) also intended to follow the advice (Abioye Kuteyi et al., 2010). In the absence of satisfaction, the tendency to follow advice (even in instances of dissatisfaction) persisted.

One study showed that physicians who demonstrated ‘attentive and respectful listening’ were able to give the perception that they ‘really cared’ for their patients; this perception also resulted in patient satisfaction (Jagosh et al., 2011). A Canadian longitudinal cohort study regarding patient satisfaction and its relationship with quality and outcomes of care after acute myocardial infarction showed that 91.7% of patients reported satisfaction with their overall care. However, this satisfaction was not associated with quality indicators for myocardial infarction and clinical outcomes (Lee et al., 2008).

### Limitations and further researches

The strength and limitations of the survey reside in some points. The present study has few limitations. First, our study sample is limited to non-probability sampling and was performed in an outpatient clinic at a university hospital, and thus, does not represent all primary healthcare clinics. Second, the six-month duration from baseline to follow-up is short.

While the results are statistically significant in terms of reductions in cardiovascular risk factors (if not with respect to the association between these declines and satisfaction with physician-patient interactions), the long-term results concerning whether target levels for BP, HDL or TC were attained cannot be assessed. The study was performed in an outpatient clinic at a university hospital, and thus, does not represent all primary healthcare clinics.

## Conclusions

The CVD risk prevention counselling based on the WHO (2007) guideline resulted in high satisfaction among moderately high-risk cardiovascular patients. However, patient satisfaction was not associated with the improvement in the modifiable cardiovascular risk factors.

##  Supplemental Information

10.7717/peerj.2983/supp-1 Supplemental Information 1 Raw data for patient satisfaction and CV risk factors

## Additional Information and Declarations

### Data Availability

The following information was supplied regarding data availability: The raw data is supplied as a Supplemental Information 1.
